# CH_4_ emissions from runoff water of Alaskan mountain glaciers

**DOI:** 10.1038/s41598-024-56608-y

**Published:** 2024-05-09

**Authors:** Keiko Konya, Tetsuo Sueyoshi, Go Iwahana, Tomoaki Morishita, Jun Uetake, Masahide Wakita

**Affiliations:** 1 Japan Agency for Marine-Earth Science and Technology (JAMSTEC), Yokohama, 236-0001 Japan; 2 International Arctic Research Center (IARC), University of Alaska, Fairbanks (UAF), Fairbanks, 99775 USA; 3 Tohoku Research Center, Forestry and Forest Products Research Institute (FFPRI), Morioka, 020-0123 Japan; 4 Hokkaido University, Field Science Center for Northern Biosphere, Tomakomai, 053-0035 Japan; 5 Japan Agency for Marine-Earth Science and Technology (JAMSTEC), Mutsu, 035-0022 Japan; 6 National Institute of Polar Research, Tachikawa, 190-8518 Japan

**Keywords:** Climate change, Cryospheric science

## Abstract

Recent studies have observed high methane concentrations in runoff water and the ambient air at various glacier sites, including the Greenland Ice Sheet, the glacier forefield in Svalbard, and the ice cap in Iceland. This study extends these findings to smaller mountain glaciers in Alaska. Methane and carbon dioxide concentrations in the ambient air near the meltwater outlet, fluxes of these gases at the surface of runoff water and riverbank sediments, and dissolved methane content in the runoff water were measured at four glaciers. Three of the four glaciers showed conspicuous signals of methane emissions from runoff water, with the Castner Glacier terminus exhibiting a methane concentration three times higher than background levels, along with elevated dissolved methane levels in the runoff water. This study marks the detection of significant methane emissions from small mountain glacier runoff, contributing to the understanding that mountain glaciers also release methane into the atmosphere.

## Introduction

As the second most important greenhouse gas (GHG) after carbon dioxide (CO_2_), methane (CH_4_) has a crucial role in future climate projections. The northern hemisphere’s high-latitude terrestrial regions are experiencing rapid changes due to amplified warming, with growing concerns regarding the acceleration of CH_4_ emissions. Earlier research has shown that subglacial environments can provide favorable temperature and pressure conditions for methanogens^1,2^, and CH_4_ production was inferred from the presence of CH_4_ oxidizers in the subglacial environments of Greenland^3^ and Antarctica^4^. Dieser et al.^3^ analyzed runoff water (RW) from Russell Glacier and detected dissolved CH_4_. Webster et al.^5^ reported elevated atmospheric CH_4_ concentrations downwind of a subglacial cave in Greenland. Wadham et al.^6^ emphasized the role of subglacial sediments beneath large ice sheets as carbon storage because of their potential impact on the global carbon cycle. In addition, a number of published papers reported high CH_4_ concentrations from glacier terminuses in Greenland and Iceland. Christiansen and Jørgensen^7^ directly measured elevated CH_4_ concentrations in subglacial air expelled from the RW, up to 15 times the background atmosphere, at glacier terminuses in Greenland. Lamarche-Gagnon et al.^8^ observed continuous runoff of methane-saturated water, suggesting that active methanogenesis may have occurred beneath the ice sheet. Burns et al.^9^ observed similar methane-saturated RWs from a temperate glacier in Iceland, Sólheimajökull.

While these studies have confirmed large CH_4_ emissions from sizable ice masses, such as the Greenland Ice Sheet and ice caps in Iceland, small glaciers have generally not been considered significant carbon sources due to their organic-poor nature, whereas ice sheets have abundant subglacial sediments^6^. CH_4_ emissions from mountain glacier margins have not been extensively observed until recent studies, with some exceptions of glaciers in Canada, in the Swiss Alps, and in Tibet^10–14^. Among them, Boyd et al.^10^ demonstrated methanogenesis in subglacial sediments, while Zhu et al.^12^ observed CH_4_ productions of thermogenic origin from the Wildstrubel Glacier in the Swiss Alps, where subglacial sediments did not have the biogenic potential to produce CH_4_. Du et al.^13^ detect the CH_4_ and CO_2_ from meltwater of high mountain glacier, Laohugou Glacier No. 12. Sapper et al.^14^ showed similar results with our study, elevated CH_4_ concentrations in the meltwater of glaciers. CH_4_ production process has also been reported for Sólheimajökull^9^. These emerging new results suggest that CH_4_ production may occur in other glaciers, including those at lower latitudes, through other processes. Although the total area covered by mountain glaciers is relatively small, they are widely distributed regardless of latitude. Depending on the conditions of GHG production, mountain glaciers could be non-negligible sources of GHGs.

**Figure 1:**
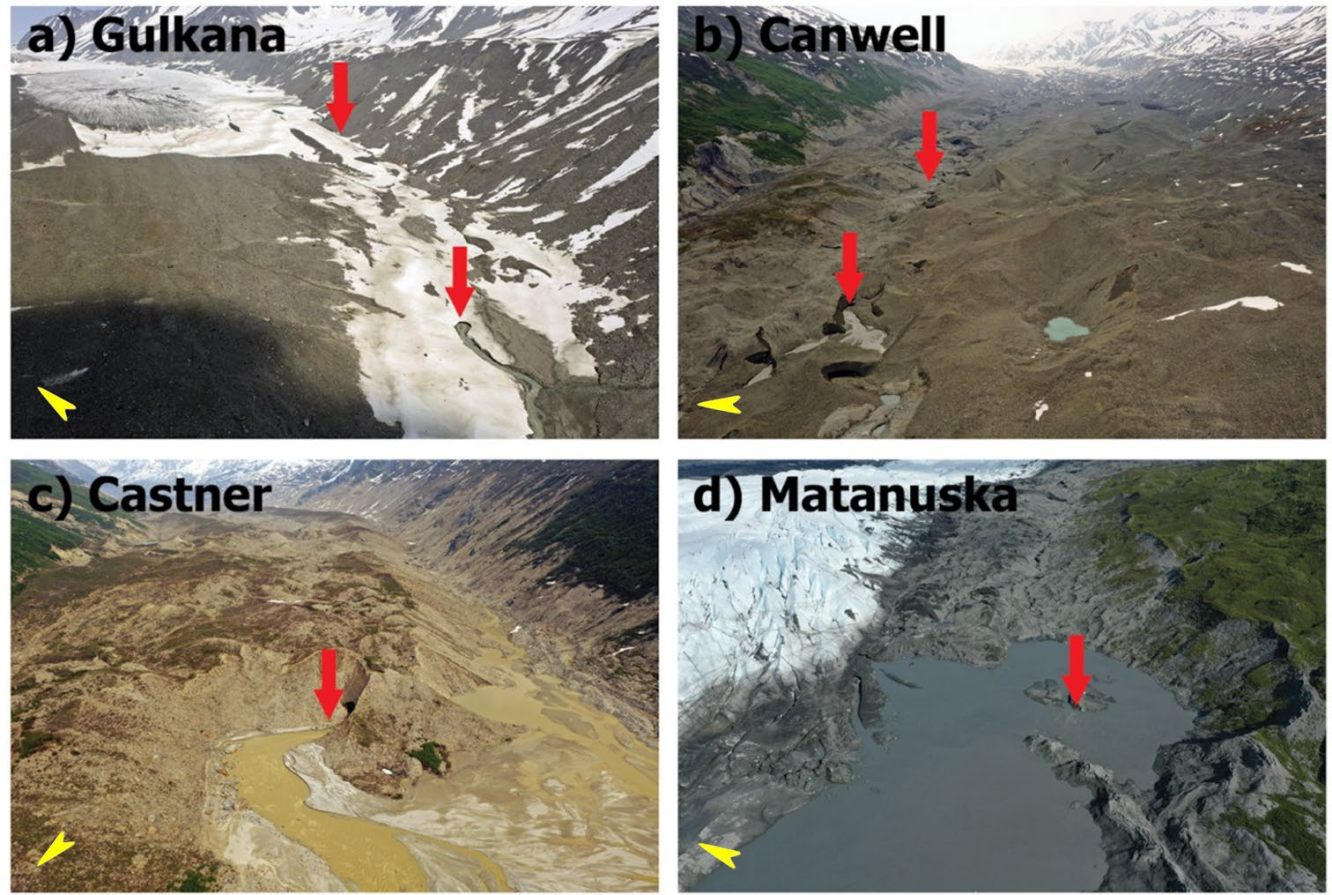
Sampling and measurement locations at the meltwater outlets at glacier terminals (red arrows). For all glaciers, the flow direction in the images is from top to bottom. Yellow arrows in the lower left corner of the panels indicate the approximate north. (**a**) Gulkana Glacier: A few outlets were found between two red arrows, near the left bank of the glacier around the terminus. (**b**) Canwell Glacier: Sampling and measurements were made at two locations close to the nearby glacier tunnel exits (within 10 m). The length of the tunnel was about 300 m, and the surface of the RW was accessible around its entrance. The downstream sampling site was 500 m away from the upstream site. (**c**) Castner Glacier: The sampling/measurements site was within 30 m of the entrance from the outlet ice tunnel. (**d**) Matanuska Glacier: Sampling and measurements were conducted within 30 m of the upwelling flow center (approximately 5 m towards the water side from the red arrow point) in the proglacial lake. Close-up images of each site can be found in the Supplementary Material (Figs. S2–S6).

Therefore, the aim of this study was to investigate whether small mountain glaciers can also emit CH_4_ through the RW. We selected four land-terminating mountain glaciers as study sites, as shown below (Fig. 1). At each glacier terminus, we measured the ambient CH_4_/CO_2_ mixing ratio above the runoff water, the CH_4_/CO_2_ flux from the adjacent surfaces (both proglacial sediment and RW), the dissolved CH_4_ in RW, and other water geochemical parameters (DOC, EC, pH).

## Results

**Figure 2:**
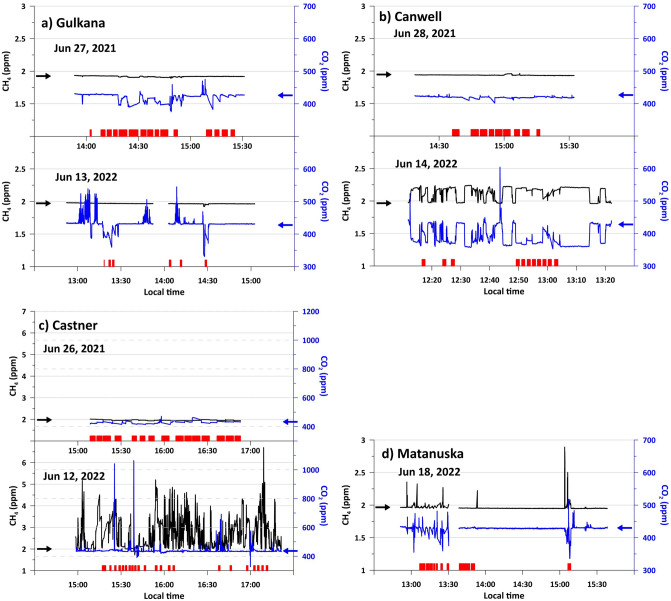
Time series of ambient CH_4_ and CO_2_ mixing ratios above the RW, measured continuously at 3-s intervals with the Picarro GasScouter G4301 at the studied glacier terminuses. The black and blue lines show the CH_4_ and CO_2_ mixing ratios (ppmv), respectively. Horizontal arrows (also in black and blue) indicate the background atmospheric concentrations of the respective gases (values of ambient air more than 30 m away from the glacier, RW) measured at each site on the same day. All glaciers except Matanuska have two panels, the upper panel for the 2021 observation and the lower panel for the 2022 observation. (**a**) Gulkana Glacier on 27 June 2021 and 13 June 2022, (**b**) Canwell Glacier on 28 June 2021 and 14 June 2022 (at the downstream sampling site), (**c**) Castner Glacier on 26 June 2021 and 12 June 2022, and (**d**) Matanuska Glacier on 18 June 2022. Note that only panel c (Castner) has a different y-axis scale, while the other three panels have a common y-axis. The red bars on the horizontal axis of each panel indicate the periods of chamber flux measurements.

In three (Canwell, Castner, and Matanusuka) of the four studied glaciers (Fig. 1), we detected elevated or decreased CH_4_ and CO_2_ mixing ratios in the air above the surface of the RW compared to the background atmospheric levels (Fig. 2).

During the 2022 observation at Castner Glacier, CH_4_ and CO_2_ mixing ratios of up to 6.7 ppm and 1060 ppm, respectively, were recorded during continuous measurements of the ambient air above the RW (Fig. 2c). We observed an increase in the CH_4_ mixing ratio when the inlet of the gas analyzer was closer to the water surface. Combined with the elevated amount of dissolved CH_4_ in Castner RW (Table 1), we interpret that the elevated level of CH_4_ is due to the expelled emission from the RW. We consider that the fluctuations (rapid ups and downs) in mixing ratios are caused by turbulence, i.e., the mixing of the subglacial air mass with the ambient air above it due to the instantaneous changes in wind direction and speed. Although the CH_4_ flux from runoff water should vary as the river water is also turbulent, the effect of air mixing is considered to be much greater. Pulse-like fluctuations were also recorded for CO_2_ mixing ratios, occasionally showing values higher or lower than the background level, although less frequently than fluctuations in CH_4_. CH_4_ mixing ratios at the Gulkana Glacier remained stable, while we observed occasional fluctuations in CO_2_ mixing ratios in both years (Fig. 2a). At the Canwell and Matanuska Glaciers, slightly elevated CH_4_ mixing ratios of up to 2.2 and 2.9 ppm, respectively, were recorded (Fig. 2b/c). At both glaciers, several pulses with high CH_4_ and CO_2_ mixing ratios were recorded at unexpected times when chamber flux measurements were not conducted. The inverse correlation between CO_2_ and CH_4_ mixing ratios can be observed in Canwell, although the range of fluctuations in mixing ratios is smaller (Fig. 2b). No clear relationship between the two gas mixing ratios was found for the other glaciers. A similar measurement was made at the same location in the previous year (26 June 2021), where little fluctuation was found in the measured gas mixing ratios (Fig. 2c).

**Table 1:** Water quality of runoff water.

Glacier	Date	Dissolved CH_4_			EC	pH	DOC		Note
		(nmol CH_4_/L)	N	SD (nmol CH_4_/L)	(mS/cm)		(µmol C/L)	N	
Gulkana	2021-Jun-27	2.8	2	0.0	0.08	7.6			
	2022-Jun-13	2.9	3	0.1	0.12	8.0			
	2022-Jul-30						17	2	
Canwell	2021-Jun-28	2.9	3	0.1	0.07	7.7			
	2022-Jun-14	5.3	3	0.1	0.15	6.6	18	3	Downstream
	2022-Jun 14	3.1	3	0.1	0.16	7.9			Upstream
Castner	2021-Jun-26	4.1	2	0.0	0.28	7.4			
	2022-Jun-12	120	3	9.5	0.36	7.6			
	2022-Jun-12						33	3	
	2022-Jul-31						23	2	
Matanuska	2022-Jun-18	5.1	6	0.1	0.17	7.9			

Dissolved CH4: concentration of dissolved CH4 in the RW, SD: standard deviation for dissolved CH4, EC: electrical conductivity, DOC: dissolved organic carbon in the RW. N: number of repeated measurements. The values of dissolved CH4 in Gulkana (2021, 2022) and Canwell (2021) are close to the detection limit. Note that the DOC samples from Castner and Gulkana in 2022 were collected separately at the end of July. The EC and pH were measured once at each site.

The dissolved CH_4_ concentrations (Table 1), shown with the standard deviation, in RW were above background levels (3.0 nmol CH_4_ L^−1^) at Castner (120 ± 9.5 nmol CH_4_ L^−1^), Canwell (5.3 ± 0.1 nmol CH_4_ L^−1^), and Matanuska (5.1 ± 0.1 nmol CH_4_ L^−1^), with Castner Glacier being the highest of the four glaciers. In addition, at Canwell, the concentration downstream of the ice tunnel (5.3 ± 0.1 nmol CH_4_ L^−1^) was considerably greater than that upstream (3.1 ± 0.1 nmol CH_4_ L^−1^).

The largest CH_4_ fluxes from the RW surface (106 µmol m^−2^ h^−1^ on average) were observed at Castner Glacier in 2022 (Table 2). The values exhibited relatively large variations ranging from 13 to 304 µmol m^−2^ h^−1^. Canwell and Matanuska Glaciers also showed positive CH_4_ fluxes (6.9 and 9.7 µmol m^−2^ h^−1^, respectively) in 2022. From sediment surfaces, positive fluxes were observed at Castner (50 µmol m^−2^ h^−1^) and Canwell (1.7 µmol m^−2^ h^−1^) in 2022. On the other hand, no significant CH_4_ fluxes were observed from any surface in 2021. Notably, CO_2_ was absorbed into the RWs at all the glaciers except Castner, where no significant CO_2_ flux was detected (Table 2).

**Table 2:** CH_4_ and CO_2_ fluxes from runoff water and sediment surfaces measured with the Picarro GasScouter G4301 and mobile chamber.

Glacier	Year	Month	Surface type	CH_4_	SD	N	CO_2_	SD	N
				(µmol/m^2^/h)			(mmol/m^2^/h)		
Gulkana	2021	Jun	Pond/surface flow water/sediment	− 0.21	0.89	12	− 0.9	1.7	12
	2022	Jun	RW	0.16	0.15	4	− 3.6	0.7	4
Canwell	2021	Jun	Sediment	− 0.10	0.13	4	− 0.1	0.2	3
	2021	Jun	Pond/surface flow water	0.39	0.56	7	− 0.5	0.6	6
	2022	Jun	Sediment	1.7	3.5	6	− 1.2	1.2	3
	2022	Jun	RW	6.9	7.3	5	− 4.8	3.2	3
Castner	2021	Jun	Sedeiment	− 0.08	0.28	12	− 0.4	0.2	12
	2022	Jun	Sediment	50	68	5	–	–	–
	2022	Jun	RW	106	141	7	–	–	–
Matanuska	2022	Jun	Sediment	− 0.05	0.07	6	–	–	–
	2022	Jun	RW	9.7	6.7	9	− 3.3	0.6	4

*SD* standard deviation of CH_4_ and CO_2_ fluxes, *N* number of locations measured. Note that the CH_4_ fluxes are shown in µmol, while the CO_2_ fluxes are shown in mmol.

Among the glaciers studied, the electric conductivity (EC) values of RW in Castner Glacier were the highest (0.28 and 0.36 mS cm^−1^ in 2021 and 2022, respectively) (Table 2). The pH of the RWs ranged from 7.4 to 8.0, with an exceptional value of 6.6 occurring at the downstream sampling site of the Canwell Glacier in 2022. The dissolved organic carbon (DOC) in the RW was greater (23–33 µmol C L^−1^) for the two sampling sites at Castner Glacier than at the Canwell and Gulkana Glaciers (~ 17 µmol C L^−1^) (Table 2). From visual observations, turbidity is also highest in Castner, which can be seen from the brown color of the RWs, as shown in Fig. 1.

## Discussion

Our observations of several Alaskan glaciers indicate that CH_4_ release from the glacier terminus (Tables 1 and 2) is not limited to large glaciers and ice sheets/caps where organic-rich sediments are more likely to survive under the ice mass^7,8,10^ but can also be found in smaller glaciers. Although the observed mixing ratios and fluxes exhibited large variability, the consistently elevated values of dissolved CH_4_ support the occurrence of CH_4_ emissions from the RWs in these glaciers. The variability in mixing ratios and fluxes can be attributed to water turbulence.

To determine the source of carbon needed to produce CH_4_, we need to examine both geological and biogenic processes. According to research on geological CH_4_ seepage^15^, four glaciers are in the region of “last glacial maximum glaciation”, and there are possibilities for seeping, faults, conventional hydrocarbons, and deposition. The Delta Mountain Glaciers is in a latitudinal zone where only the formation and release of modern CH_4_ can occur, while Matanuska is in a latitudinal zone where seeping emission of geologic CH_4_ can occur. A recent study in Svalbard^16^ also documented that CH_4_-rich groundwater can be formed during the retreat of glaciers by nonmicrobial processes. Another case in Iceland showed that geothermal effects can also explain biogenic production^9^. If the process is biogenic, there must be sufficient organic matter or carbon in the glacier bed for methanogenic bacteria to produce high concentrations of CH_4_, which is subsequently dissolved in the glacier meltwater^17^. We suggest three possible candidates for the supply of carbon to the beds of observed mountain glaciers, the first two of which were also suggested for Greenland by previous studies^7,8^. (1) Organic carbon supplied from the glacier surface: Cryospheric microorganisms on the glacier surface could be the main source of organic carbon. They exist even on the Gulkana Glacier (clean type glacier)^18^, and debris-covered glacier surfaces are more suitable environments for microbial activity. (2) Old subglacial sediments: Peat or other carbon-rich sediment, which developed during a past glacial retreat, could be preserved in the glacier bed. (3) Supply from glacial bedrock: The bedrock beneath the studied glaciers is mainly metamorphic sedimentary rock, such as phyllite and schist from Cretaceous or older ages^19^, which could have originated from organic-rich sedimentary rocks. Methanogenesis in deep organic-rich sedimentary rocks is known^20,21^ and is one of the possible CH_4_ production mechanisms under anaerobic conditions beneath glaciers.

Unfortunately, the observations are too limited to narrow down the list confidently. Instead, we suggest the following approaches as the next steps to address this issue: (1) ^14^C dating of DOC and POC and, if possible, of dissolved CH_4_ itself. (2) Stable isotope analysis of CH_4_ from different glaciers in different seasons to determine the key process and whether multiple processes are involved. (3) Direct sampling of subglacial sediment, which could be possible via hot water drilling or ice coring. Among these possibilities, the combination of ^14^C dating and isotope analysis is considered an effective approach because it strongly constrains potential processes and is cost effective.

Although the observation period was very limited and our data represented only snapshots of emissions from glacial RWs, the measured CH_4_ fluxes were not small in comparison with those from other ecosystems. According to the Global River Methane Database^22^, the global median CH_4_ flux from rivers is 18 µmol m^−2^ h^−1^, which is one order of magnitude lower than the observed CH_4_ flux from the runoff water of Castner Glacier (106 µmol m^−2^ h^−1^); however, these fluxes will not contribute to global emissions, as the water surface around the glacier terminus is negligibly smaller than the global river area. According to our comparisons with Arctic lakes^23,24^, the flux from Castner is greater than the flux from non-yedoma lakes (ca. 45.8 µmol m^−2^ h^−1^), although it is smaller than the flux from continuous yedoma lakes (ca. 358 µmol m^−2^ h^−1^). However, the total emissions are not comparable because the area of the thermokarst lakes is several orders of magnitude larger than the water surface around the glacier terminus. Nevertheless, our results indicate that mountain glaciers can be important local CH_4_ sources.

The impact on the global CH_4_ budget needs to be discussed with additional data. From our observations of the Alaskan glaciers, it is not clear whether the emissions from a glacier are large enough to affect the global CH_4_ budget. As there are large differences between glaciers and diurnal and seasonal variations are also likely to be large, additional observations should be made on more glaciers from different areas and throughout the melt season to verify their temporal and spatial variability.

## Materials and methods

### Study site

In light of the purpose of this study, we chose mountain glaciers in the Arctic from an area not showing geological/volcanic activity and not underlain by ice-rich permafrost, hopefully permafrost free. Glaciers should be moderately retreated in recent decades and not exhibit special characteristics, such as surge-type. They should be easily accessible, which is important for repeated measurements. These criteria were used to select glaciers in the Delta Mountains. Matanuska Glacier was added to cover the different types of glaciers.

**Figure 3:**
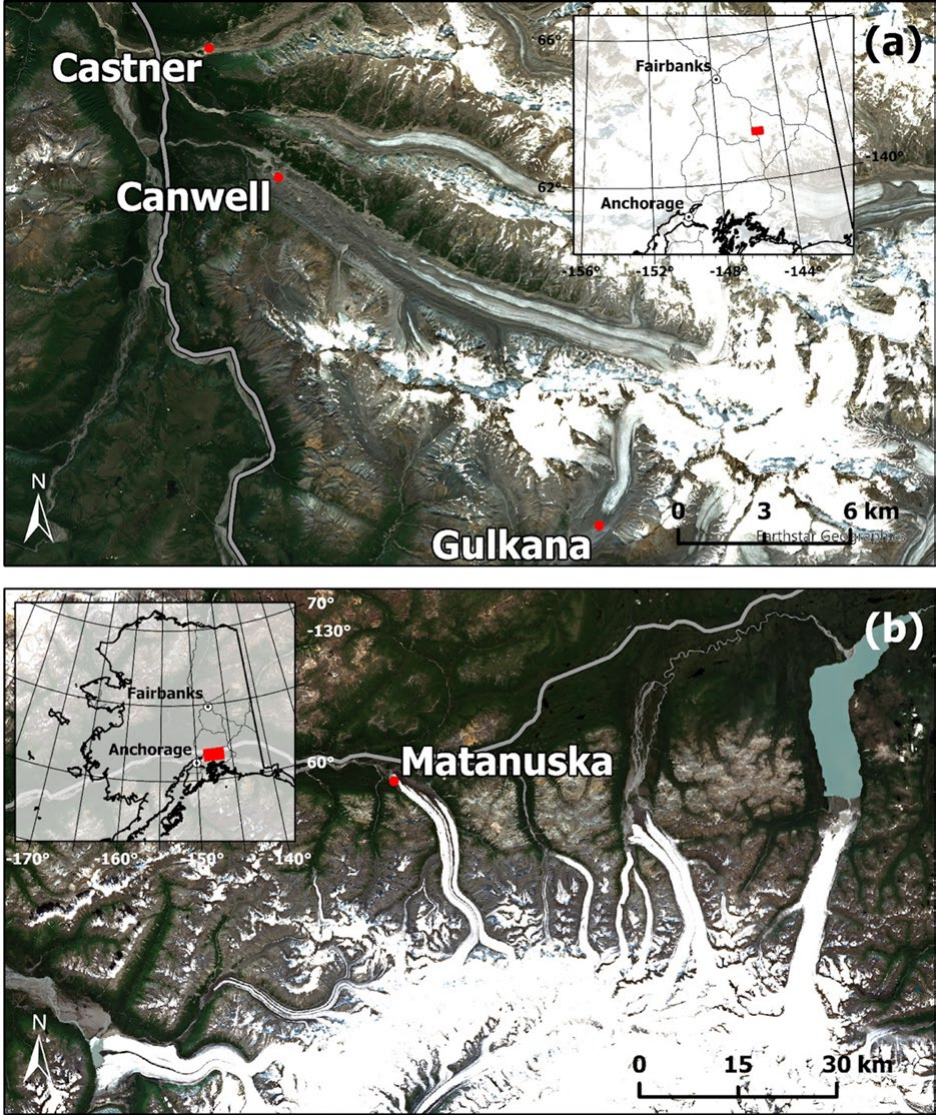
Locations of the studied glacier terminals (red dots). (**a**) Castner, Canwell, and Gulkana Glaciers accessed from the Richardson Highway; (**b**) Matanuska Glacier accessed from the Glenn Highway. Source of satellite images: Google Earth (Accessed: 5th May 2023).

All four glaciers have been retreating during recent decades. They have different physical characteristics and are suitable for comparison with each other (Table 3). Three of them (the Castner, Canwell, and Gulkana Glaciers) are located in the Delta Mountains, an eastern section of the Alaska Range (Fig. 3a). The Matanuska glacier is located near Anchorage in southern Alaska (Fig. 3b). Gulkana Glacier (63.25° N, 145.42° W) is a polythermal glacier with an elevation of 1160–2470 m above sea level, a length of 8.6 km and an area of 16.0 km^2^ that shrank by 14% between 1967 and 2016^25^. Meteorological and glaciological observations, including mass balance measurements, were conducted by the USGS. Castner Glacier (63.41° N, 145.68° W) is 19.6 km in length, and more than half of its area is covered by debris. Canwell Glacier (63.33° N, 145.52° W) is 23.5 km in length and covered by debris around the terminus. Matanuska Glacier (61.77° N, 147.75° W) is 43.2 km long, 3.2 km wide, and a clean glacier with a small lake at the terminus (Table 3). For all four glaciers, despite the difference in surface debris coverage, the development of supraglacial channels was not visible in the drone images (Fig. 1). Meltwater drainage is considered to occur mostly through subglacial systems.

**Table 3:** Glacier specifications.

Glacier	Area (km^2^)	Length (km)	Elevation range (m)	Average slope angle	Type	Lon, Lat	Geology
Gulkana	17.567	8.6	1341–2120	14°	Clean	145.43 W, 63.257 N	Metamorphic, volcanic, and sedimentary
Canwell	59.601	23.5	808–2734	14°	Debris covered	145.62 W, 63.379 N	Schist and phyllite
Castner	53.795	19.6	787–2941	17°	Debris covered	145.69 W, 63.406 N	Schist and phyllite
Matanuska	308.689	43.2	1744–1837	16°	Clean	147.75 W, 61.773 N	Accretionary complex (sandstone and shales)

Geographical information for the glaciers is obtained from the Randolph Glacier Inventory^26,27^. Castner and Canwell Glaciers are covered with debris in the ablation area and are classified by the authors as “debris covered”. Observation points are given in longitude and latitude. Geological information is taken from the USGS geological map^19^.

Regarding the geological settings, the glaciers in the Delta Mountain developed over schist and granitic rocks, while the Matanuska glacier is underlain by sandstone and shale with some metamorphism^19^ (Fig. 3).

The mean annual air temperature at Paxson, 19 km south of the Gulkana Glacier, was − 1.7 °C, and the mean annual precipitation was 342 mm, while in Anchorage, it was 3.6 °C and 481 mm, respectively, during 2019–2022^28^. Although permafrost is distributed in the surrounding mountains, the bottom of the valley is not underlain by permafrost, and no yedoma ice complex is distributed for any of the glaciers^29^.

We conducted measurements and sampling activities within 30 m of the outlet of each glacier terminus. Except for the Canwell Glacier, our measurement and sampling locations were the first locations where glacier outflow occurred, as far as we could judge from our drone surveillance. The terminal area of the Canwell Glacier was extensive, and the outflow streams were intermittently open to the air or under glacial tunnels. At Canwell Glacier in 2022, our research was conducted at two locations close to nearby glacier tunnel exits (within 10 m). The length of the tunnel was approximately 300 m and the distance between the two locations was approximately 500 m (red arrows in Fig. 1b). The outflow was mostly open more than 1 km upstream of the upstream sampling location. The measurements and sampling in 2021 were conducted approximately 100 m downstream of the downstream sampling location in 2022, with different configurations of outflow streams and glacier tunnels.

### Design of the measurement

The aim of the field campaign was to cover several glaciers with some similarities and differences so that we could compare the results rather than focusing on one glacier to observe temporal variations. To do this, we took one site per glacier at the main outlet. For comparison, a series of observations were made at similar times of day to avoid the effect of diurnal variations, which was reported in one of the earlier studies in Greenland^8^. As there is a variation in the amount of RW, it is reasonable that diurnal variation existed. The observation period was kept rather short to avoid seasonality, as we could not carry out the simultaneous observation campaign in several glaciers in parallel. The field campaigns were carried out during the early ablation season, 26–28 June 2021 and 12–14 June 2022.

At each site, we planned the measurements/sampling of the following items. (1) GHG mixing ratio: ambient air above the runoff water; (2) GHG mixing ratio: background atmosphere; (3) GHG flux on the riverbank sediment; (4) GHG flu x on the water surface; (5) EC/pH measurement on runoff water; (6) sampling of runoff water for dissolved CH_4_ measurement (headspace method); and (7) sampling of runoff water for DOC analysis. A portable gas analyzer (Picarro GasScouter G4301) was used for steps (1)–(4), and mobile water quality sensors (HORIBA B-771 and B-712) were used for step (5). A description of the gas analyzer and mobile sensor is given in the following sections.

The measurement procedures for steps (1)–(7) were as follows: (1) The gas analyzer was located at the edge of the riverbank, facing the runoff stream. The air inlet of the gas analyzer was fixed at 10 cm high, at the exit of (Canwell, Fig. S3), or 5 m away from (Castner, Fig. S4), the ice tunnel. The inlet was fixed at the same height on the meltwater outlets at the glacier surface (Gulkana, Fig. S2) or at the riverbank 30 m away from the upwelling point (Matanuska, Fig. S5). Measurements were conducted continuously for approximately one hour. (2) The gas analyzer was located more than 30 m from the runoff water surface. The air inlet of the gas analyzer was fixed at a height of 1 m. Measurements were taken until the observed values stabilized, usually after approximately 15 min. (3) More than three measuring points on riverbank sediment were selected around the glacier terminus. CH_4_/CO_2_ fluxes were measured using a gas analyzer and a mobile chamber. The measurement time was at least 60 s. (4) The mobile chamber of the gas analyzer was manually placed on the water surface and held at least 60 s for the flux measurements. CH_4_/CO_2_ flux measurements were repeated more than three times. (5) RW samples were taken from the riverbank, and EC and pH were measured using mobile sensors. (6) RW samples were taken from the riverbank, and following the headspace method procedure, air samples were collected in prevacuumed vials using syringes and three-way stopcocks. (7) RW samples were taken from the riverbank and collected in prepared glass bottles.

### Field-portable gas analyzer for CH_4_ and CO_2_ mixing ratios and fluxes

The mixing ratios of CH_4_ and CO_2_ in the ambient air and in the mobile chamber were measured with a GasScouter G4301 (Picarro, Inc.) cavity ring-down spectroscopy (CRDS) gas concentration analyzer with a dedicated mobile flux chamber (floor area: 500 cm^2^, volume: 5000 cm^3^). The instrument used was calibrated by the manufacturer prior to our measurements. The detailed specifications of the device are provided in the Supplementary Material and available online^30^. The precision of the G4301 measurements is less than 0.15 ppm for CO_2_ and 0.8 ppb for CH_4_. The measurement interval was 3 s, and the response time was 5 s. Usage at the observation site with images is also shown in the Supplementary Material.

The surface gas flux was calculated from the rate of change of the gas mixing ratio in the chamber using linear regression. The mixing ratio time series was trimmed so that only the monotonically changing part was used for the flux calculation. Due to this procedure, the measurement time for the flux calculation ranged between 34 and 143 s, shorter than the original measurements (> 60 s). For the flux at the water surface, several measurements revealed multiple increases and decreases in the gas mixing ratio; these changes were considered failures due to the incomplete closure of the chamber to the water surface. We used only time series with monotonic changes (R^2^ > 0.6) or small fluctuations (< ± 1.0 CH_4_ µmol m^−2^ h^−1^, < ± 0.1 CO_2_ mmol m^−2^ h^−1^) to avoid erroneous flux estimation. Details of the flux calculation procedure is available in the Supplementary Material.

### Headspace method for dissolved CH_4_

The dissolved CH_4_ concentration in the water was measured and calculated according to methods described in previous works^31–33^. A 50 ml water sample was taken into a 100 ml plastic syringe with a three-way stopcock, 50 ml of ambient air was immediately introduced into the syringe, and the stopcock was closed. Then, the syringe was shaken vigorously by hand for more than three minutes, and a 40-ml air sample from the headspace was taken into a 30-ml vacuum glass bottle with a butyl rubber stopper and a plastic cap. Then, only the air left in the syringe, not water, was removed from the syringe, 50 ml of air was returned to the syringe, and the same procedure was repeated. Namely, we extracted twice from each sample. We then calculated the total amount of CH_4_ from the sum of the two extractions for each sample and subtracted the background value (approx. 2 ppm) from the equilibrated headspace concentration, as we did not use N_2_ gas for these measurements. Ambient air samples were also taken at each site for this purpose (to measure the background level).

All operations were performed at each study site, all the samples were brought to Japan, and the CH_4_ concentration was determined in the laboratory of FFPRI using a gas chromatograph according to the method described in Konya et al.^34^. The minimum detectable dissolved CH_4_ concentration in this study was 2.8 nmol CH_4_ L^−1^.

### Measurement of water geochemistry (EC, pH, and DOC)

The EC and pH of the sampled water were measured at the study sites using B-771 and B-712 mobile sensors (HORIBA, Kyoto, Japan), respectively. Calibration of the EC and pH meters was conducted daily. Standard solutions with a pH of 4.01/6.86 and an EC of 1.413 mS/cm (at 25 °C) were used for the pH and EC meter measurements, respectively. Water samples for DOC measurements were collected directly from the outflow stream into clean plastic bags (Whirl–Pak, Nasco, WI, USA) and kept frozen at -35 °C during transportation to the laboratory. Melted samples were transferred to two or three 60 ml high-density polyethylene (HDPE) bottles. The collected HDPE bottles were frozen upright and maintained at less than − 20 °C until analysis. After a sample was returned to room temperature for analysis, it was acidified to pH < 2 with hydrochloric acid and bubbled with carrier gas (high-purity air) to remove dissolved inorganic carbon. The DOC concentration was determined by using a total organic carbon analyzer (TOC-L, Shimadzu Co., Kyoto, Japan) according to the analytical method of Wakita et al.^35^. The precision for the DOC concentrations was ± 0.8 µmol C L^−1^, where the number indicates the standard deviation of the absolute values of the differences between samples. The detection limit was 1.2 µmol C L^−1^.

### Supplementary Information

Supplementary Information.

## Data Availability

The dataset of CH_4_ and CO_2_ mixing ratios is presented in Fig. 2, and the original values used to calculate the mean values are available in Tables 1 and 2 from the ADS data repository (https://ads.nipr.ac.jp/dataset/A20230802-001).
