# Supplementary material for: CH4 emissions from runoff water of Alaskan mountain glaciers
Source: Sci Rep. 2024 May 9;14:10558. doi: 10.1038/s41598-024-56608-y (PMC11082196; doi:10.1038/s41598-024-56608-y)
Supplement: Supplementary file 1 — Supplementary Information. [file 41598_2024_56608_MOESM1_ESM.pdf]

Supplementary Information to

CH_4 emissions from runoff water of Alaskan mountain glaciers

Konya, K[1]; Sueyoshi, T[1,*]; Iwahana, G[2]; Morishita, T[3]; Uetake, J[4]; Wakita, M[5]

[1]*Japan Agency for Marine-Earth Science and Technology (JAMSTEC), Yokohama, 236-0001, Japan*

[2]*University of Alaska, Fairbanks, International Arctic Research Center (IARC), Fairbanks, 99775, USA.*

[3]*Tohoku Research Center, Forestry and Forest Products Research Institute (FFPRI), Morioka, 020-0123, Japan*

[4]*Hokkaido University, Field Science Center for Northern Biosphere, Tomakomai, 053-0035, Japan*

[5]*Japan Agency for Marine-Earth Science and Technology (JAMSTEC), Mutsu, 035-0022, Japan*

* Corresponding author.
Phone. +81 (0) 45 778 5539.
E-mail address: sue@jamstec.go.jp / sueyoshi.tetsuo@gmail.com

1. Supplementary information on the study sites

1-1. Locations of the glaciers

Information on the rough location. Delta Mountain glaciers in the Alaskan range and Matanuska Glacier are shown on a map of the State of Alaska. The criteria for choosing the observation sites were as follows: 1) the area is not affected by volcanic activity, 2) the area is not underlain by ice-rich permafrost, 3) the glaciers moderately retreated in recent decades, 4) the glacier does not exhibit special characteristics (e.g., surge type), and 5) the glacier has a reasonable accessibility.

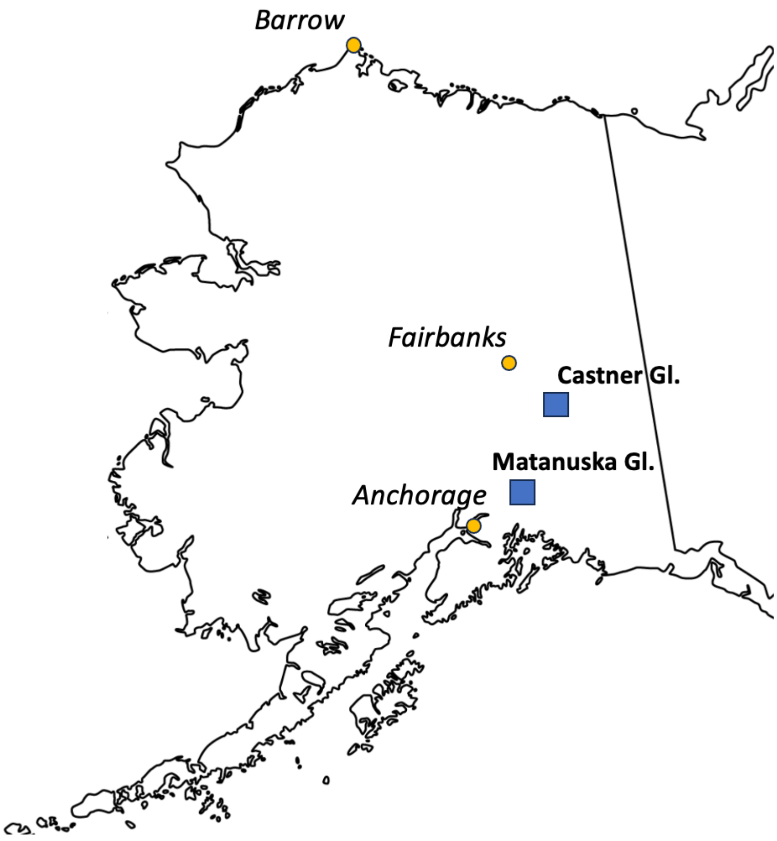

Supplementary Figure S1: Location of Castner Glacier (Delta Mountain glaciers) and Matanuska Glacier.

1-2. Close-up photos of Sampling sites

Figures S2-S6 show close-up images of four glaciers at each sampling site: Gulkana, Canwell, Castner, and Matanuska. All the sampling/measurement sites are within 30 m from the outlet of the glacier runoff water.

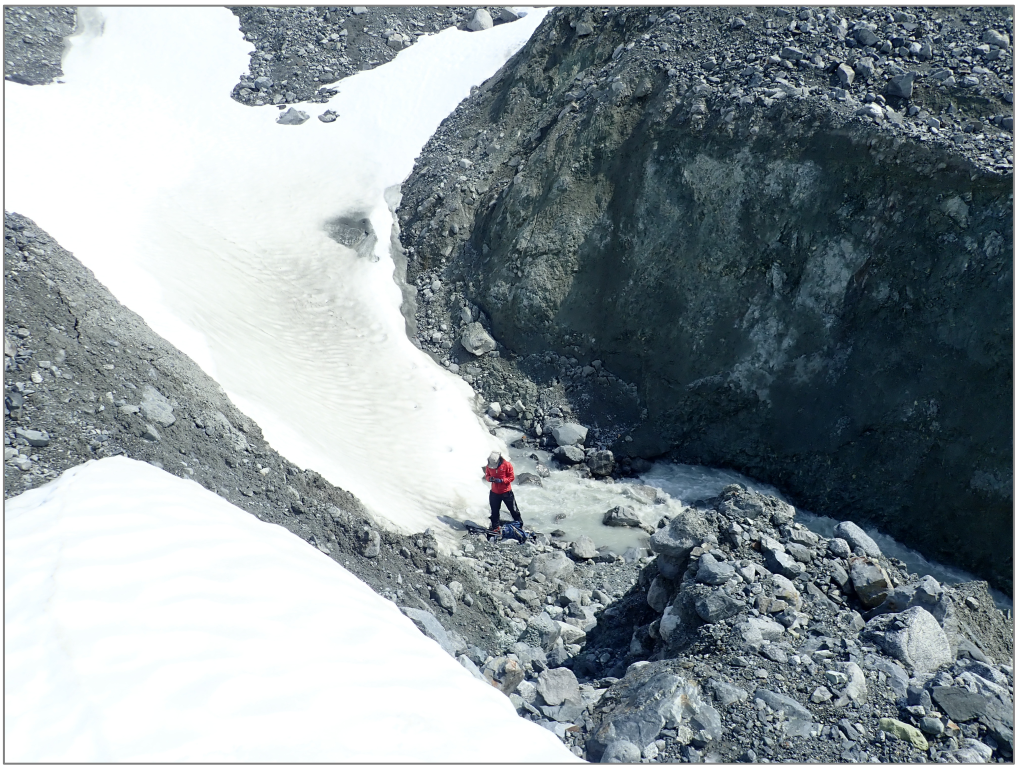

Supplementary Figure S2: Sampling site of the Gulkana glacier. A meltwater outlet was formed on the left margin, close to the left bank of the glacier. The runoff water flows along the left bank.

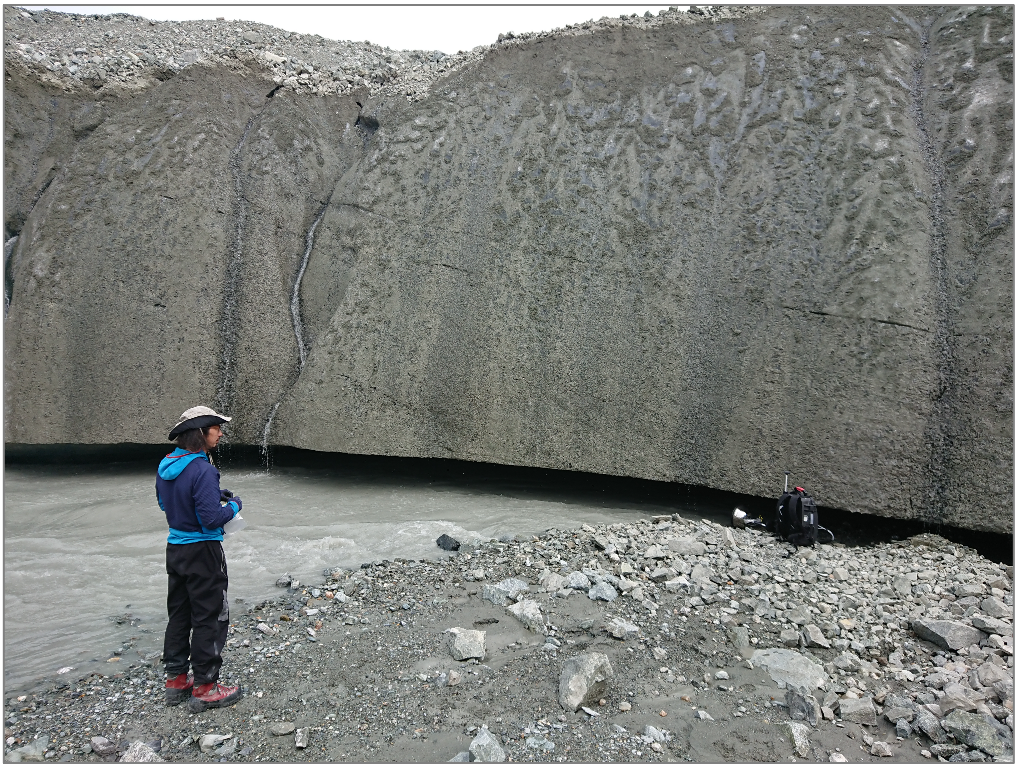

Supplementary Figure S3: Sampling site on Canwell Glacier. Meltwater flows out from under the glacier ice.

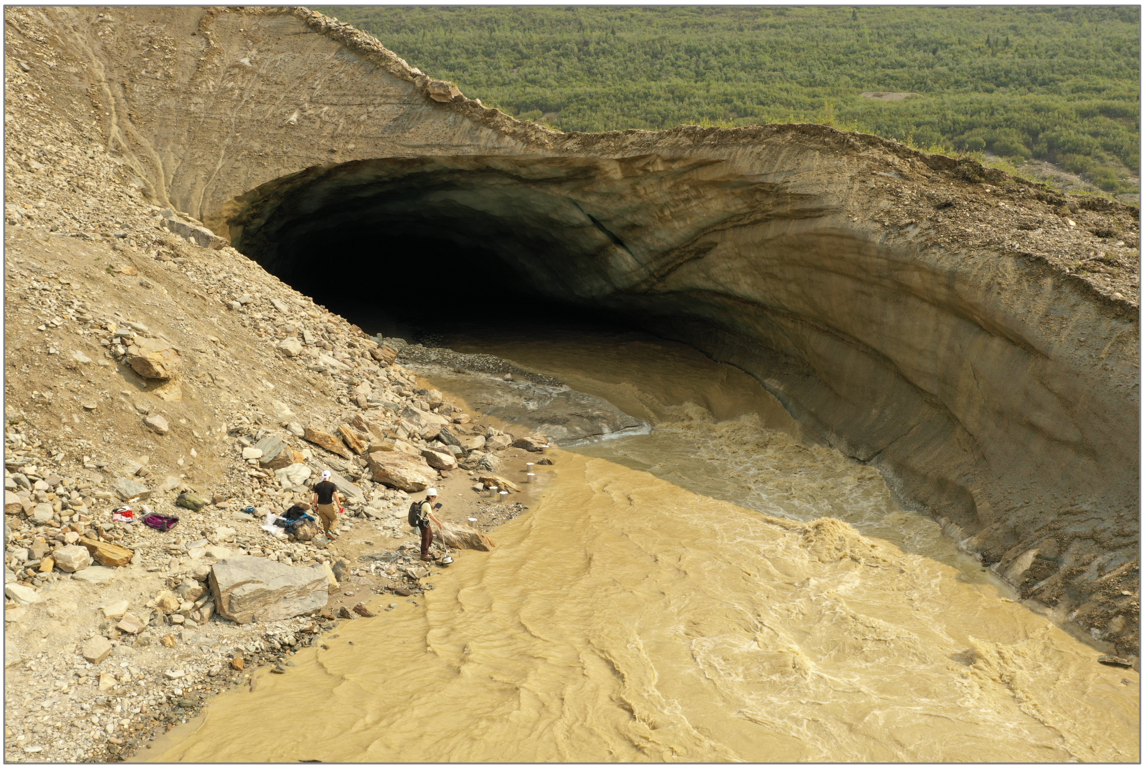

Supplementary Figure S4: Sampling site on Castner Glacier. There is a large ice cave at the terminus of the glacier, where the runoff water runs out.

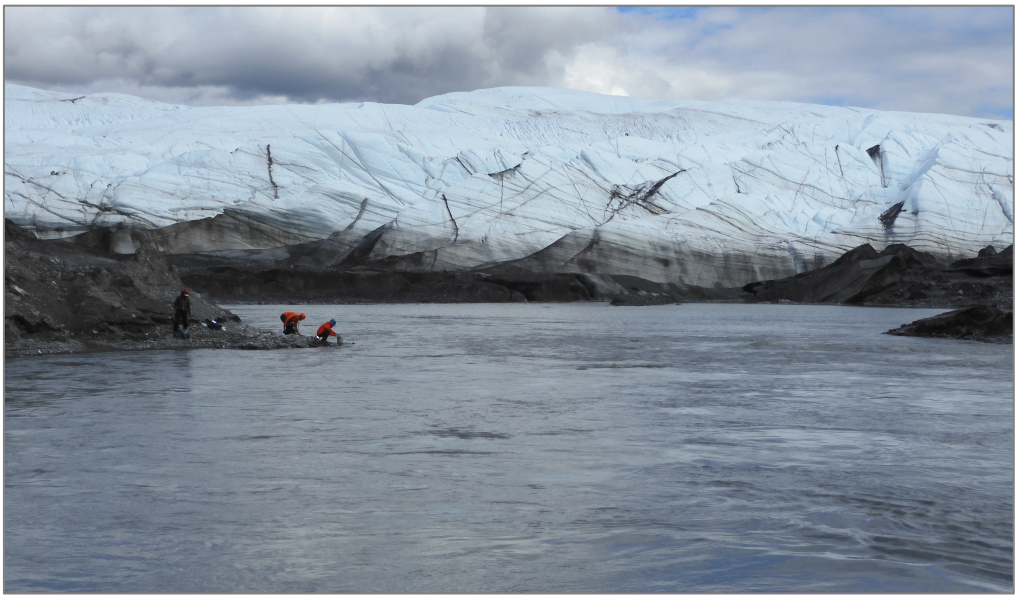

Supplementary Figure S5: Sampling site of the Matanuska Glacier. A proglacial lake formed at the terminus. Sampling and measurements were performed from the right bank, where the upwelling flow of glacial meltwater is visible in the lake.

2. GHG measurements using Picarro GasScouter G4301

2-1. Picarro GasScouter G4301

We deployed Picarro G4301, a portable device to measure the GHG mixing ratios in the ambient air and the surface flux of GHGs. The specifications of the Picarro GasScouter G4301 are shown in Supplementary Table S1 (below). This device measures the mixing ratio of the gas (CH_4, CO_2, and H_2O) introduced from the gas inlet with cavity ring-down spectroscopy (CRDS), a type of optical spectroscopic technique. The surface gas flux is measured using a connected chamber by measuring the change ratio of the gas concentration.

Specification	CO_2	CH_4	$H_2$0
**Raw precision** (5 sec)	0.4 ppm + 0.1% of reading<br>Typical = 0.15 ppm*	3 ppb + 0.1% of reading<br>Typical = 0.8 ppb*	100 ppm + 5% of reading
**Precision** (300 sec, 1σ)	0.04 ppm + 0.02% of reading<br>Typical = 0.025 ppm*	0.3 ppb + 0.02% of reading<br>Typical = 0.1 ppb*	10 ppm + 5% of reading
**Lower Detection Limit** (300 sec, 3σ)	0.12 ppm<br>Typical = 0.075 ppm*	0.9 ppb<br>Typical = 0.3 ppb*	–
**Drift** (24 hr, peak-to-peak 50 min average)	0.5 ppm<br>Typical = 0.18 ppm*	1 ppb<br>Typical = 0.56 ppb*	–
**Measurement Range**	0–3%	0–800 ppm	0–3% (non-condensing)
**Measurement Interval**	3 sec		
**Response Time** (Rise/Fall Time 10–90%/90–10%)	5 sec		

Supplementary Table S1: Data from GasScouter™ G4301 Analyzer Datasheet [1]. *Typical performance is defined as the median of testing results from 29 sequentially built G4301 analyzers measured at ambient concentration levels.

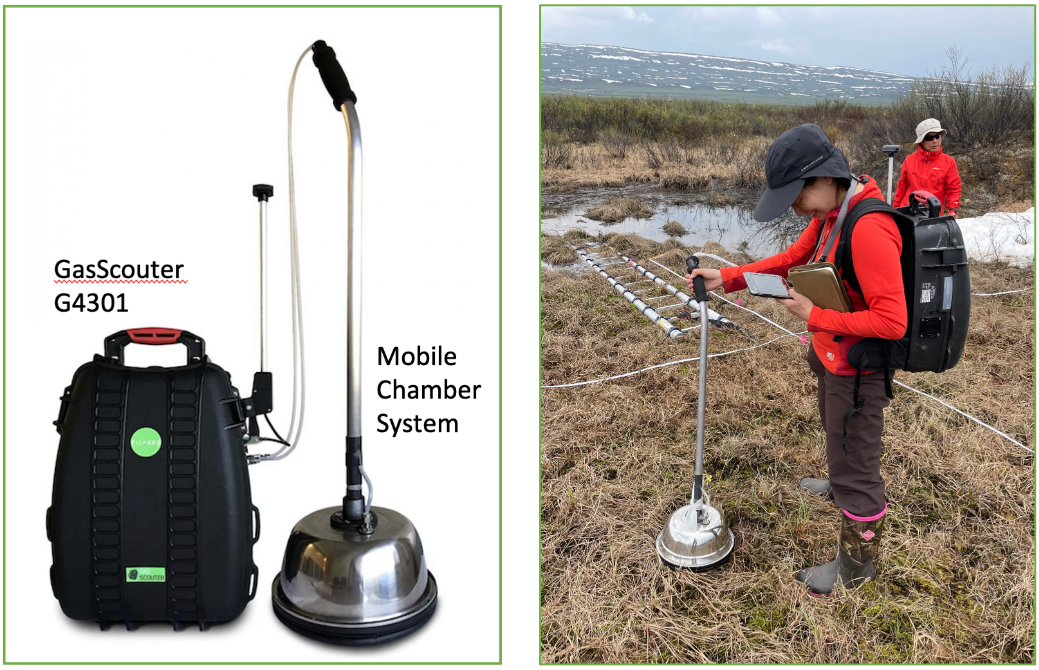

Supplementary Figure S6: Picarro GasScouter G4130 instrument with a dedicated mobile chamber (left panel) and its use at the field site to measure the flux at the ground surface.

2-2. Measurement of GHG mixing ratios

GHG mixing ratios (concentration in the air) was measured using the GasScouter G4301. The mobile chamber was used as the gas inlet, as the tube was connected to the inside of the chamber (Figure S7, left panel). The chamber and GasScouter were fixed on the ground, and the mixing ratio was continuously measured for approximately one hour.

For the measurement of the background mixing ratio, the measuring point was at least 30 m from the surface of the glacier runoff, and the gas inlet (chamber) was fixed at a height of 1 m.

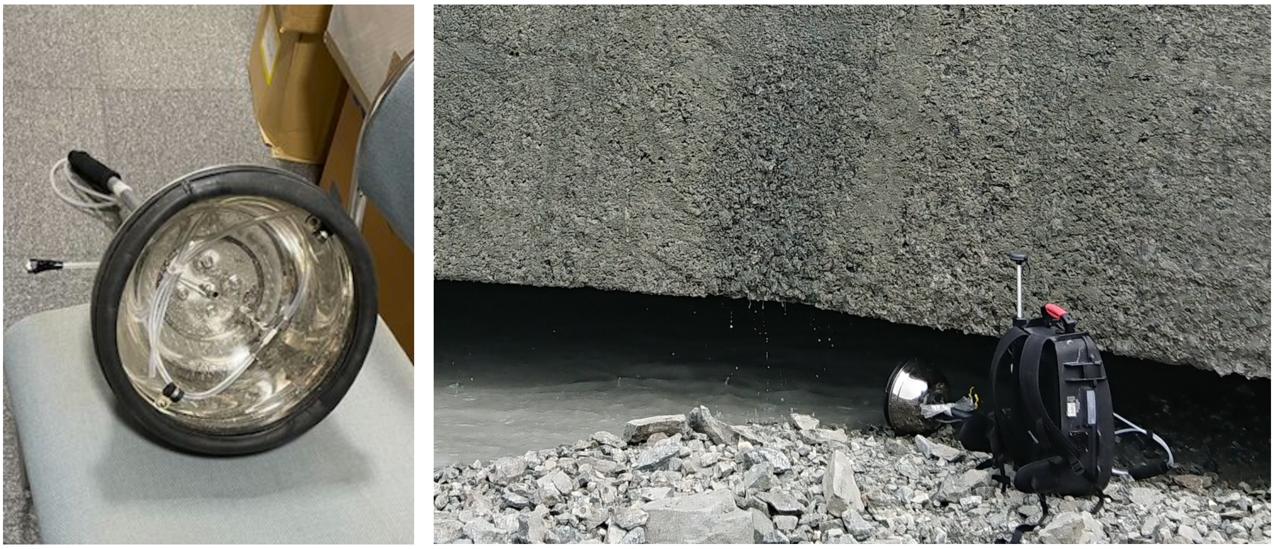

Supplementary Figure S7: The inside of the mobile chamber and the gas inlet (tube), which is connected to the GasScouter through the lid (left). Additionally, see Figure S6 for the connection of the tube between the chamber and the gas analyzer. An example of GHG mixing ratio measurements at Canwell Glacier in 2022 (right panel). The distance between the chamber inlet and the measuring column of the gas analyzer was approximately 2 meters.

2-3. Flux calculation using the chamber gas concentration

The surface flux was calculated from the rate of change in the gas mixing ratio in the chamber using the following equation:

$$F = \rho \times \triangle c / \triangle t \times V/A \qquad (1)$$

where F (g C m^{-2} s^{-1}) is the gas emission rate (flux); ρ is the density of the gas under standard conditions (CH_4: 0.716 kg m^{-3}, CO_2: 1.977 kg m^{-3}); V and A are the volume (m^3) and base area (m^2) of the chamber, respectively, where V/A is the height of the chamber from the ground surface; and $\triangle c/\triangle t$ is the rate of change in the gas mixing ratio in the chamber, which is estimated using linear regression. The specifications of the mobile chamber used in this study were V=5000 cm^3 and A=500 cm^2.
We calculated the gas densities assuming a constant chamber temperature of 10 °C at 1 atmosphere.

Flux measurements on the running water (glacier runoff water)

In the flux measurement at the surface of runoff, we found that large fluctuations in the time series can cause problems in flux calculations. Although the change in the mixing ratio is expected to be monotonic (e.g., Figure S8) as the rate of change is used in the flux calculation, we observed two types of variations for which the rate of change is difficult to calculate; and therefore, a reasonable estimation of the flux is not possible: (1) multiple rises and falls of the gas concentration during the measurement (e.g., Figure S9, left panel), which is probably due to incomplete closure of the chamber with the water surface, and (2) abrupt changes in the gas concentration when the chamber is placed on the water surface, caused by large and unstable emissions from the runoff water. For some measurements, the time series of the concentrations used for the flux calculations contained abrupt changes, especially at the beginning of the measurements (e.g., Figure S9, right panel). These high-frequency or abrupt changes occurred at the surface of the runoff water. We considered that this happened because the runoff water was flowing (moving) underneath the chamber, its gas concentrations were changing, and because of the water surface ripples.

Removal of unsuccessful measurements and the abruptly changing part

For the first (1) type of the problematic time series, we set the following criteria to distinguish successful and unsuccessful measurements: a concentration time series with monotonic changes (R2 > 0.6) or small fluctuations (<±1.0 CH_4 µmol/m2/h, <±0.1 CO_2 mmol/m2/h). The time series with multiple increases and decreases were discarded by these criteria. For the second (2) type, we examined the time series for each calculation, trimmed the problematic parts, and then recalculated each flux measurement. As a result, the measurement durations for the flux calculations become shorter than those of the original design (> 60 seconds), ranging from 34 to 143 seconds.

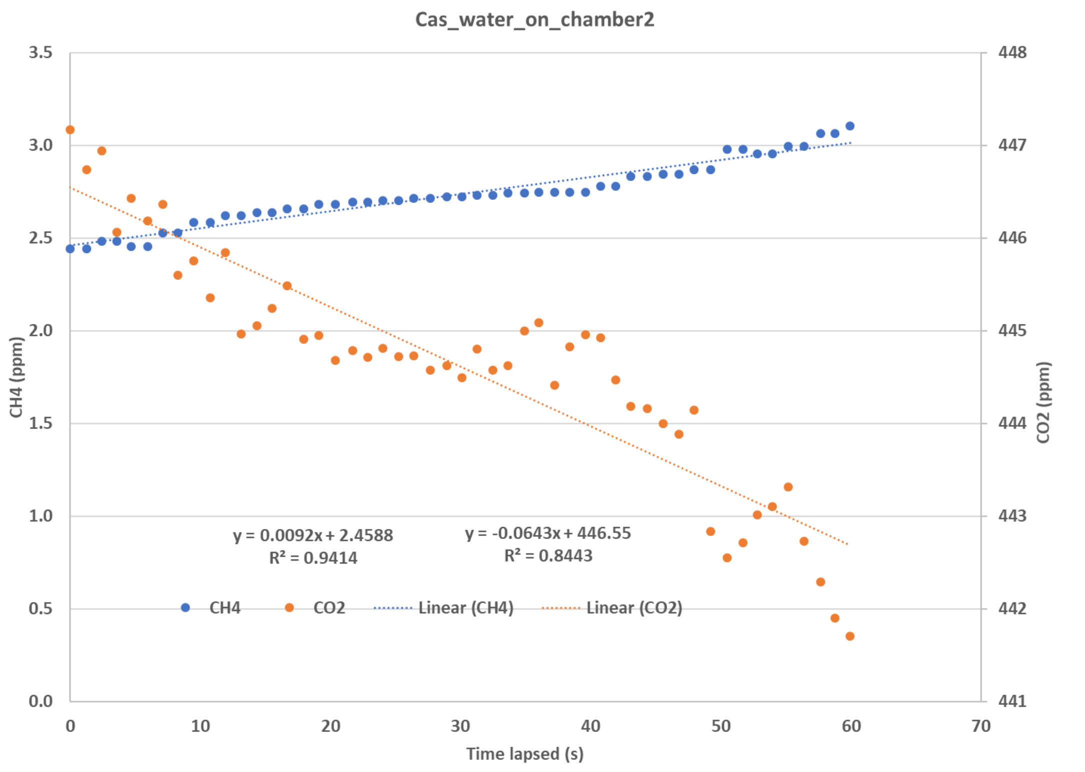

Supplementary Figure S8: An example of successful chamber measurements at Castner Glacier in 2022.

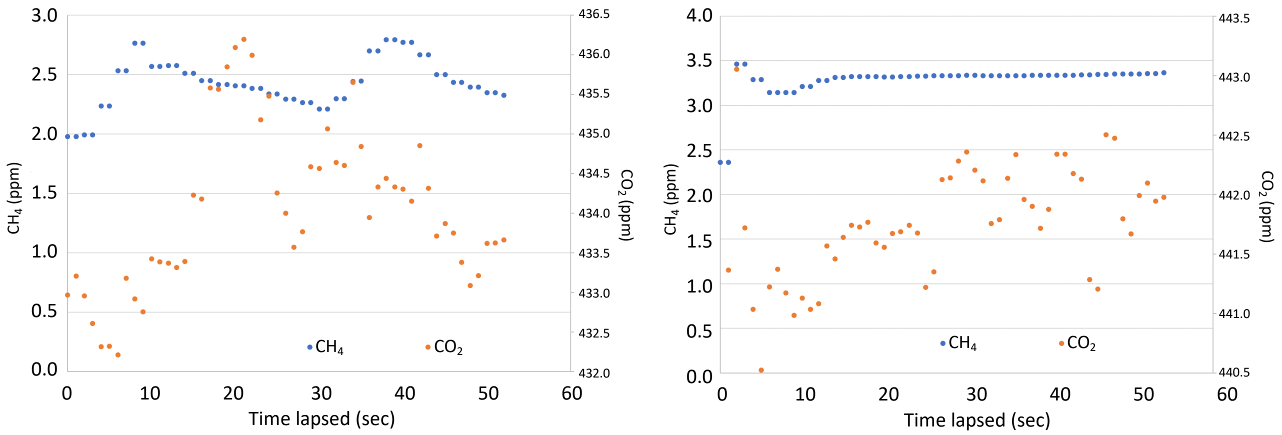

Supplementary Figure S9: Examples of unsuccessful chamber measurements. (1) Multiple increase and decrease (left panel), and (2) abrupt changes at the beginning of the measurement (right panel). Measured at Castner Glacier in 2022.

3. Sampling methods

3-1. Air sample for Dissolved CH_4 analysis (Headspace method)

Dissolved CH_4 was analyzed by the headspace method, described by McAullife [2] and Sawamoto et al. [3]. With this method, we can collect air samples from the sampling site to the laboratory without possible changes in CH_4 concentrations in the sample bottle before analysis.

At each site, a 50-mL water sample was taken with a 100-mL plastic syringe, and 50 mL of air was immediately drawn into the syringe. Then, the syringe was vigorously shaken by hand for 3 min, and a 20-mL air sample from the headspace was taken into a 10-mL vacuum glass bottle [4]. Three replications were applied.

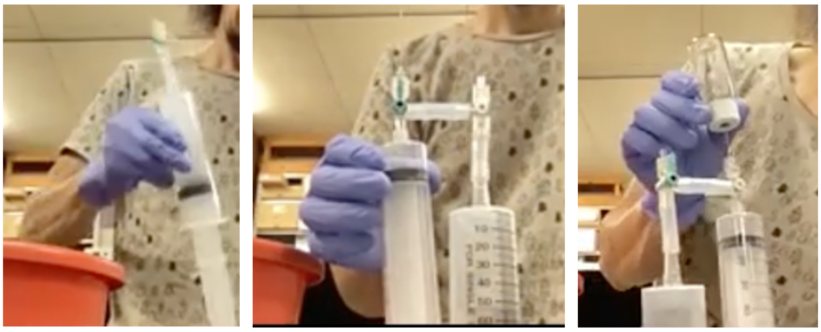

Supplementary Figure S10: Procedure of the headspace method reproduced in the laboratory. The syringe was vigorously shaken to expel the dissolved gas into the air (left), the air was moved into the connected dry syringe (middle), and the air sample was taken into the vacuum glass bottle (right).

3-2. Water samples for DOC and isotope measurements

Water samples were taken for the purpose of analyzing dissolved organic carbon (DOC) and stable isotopes. Samples for DOC measurements were collected at the riverbank directly from the outflow stream into clean plastic bags (Whirl-Pak, Nasco, WI, USA) and kept frozen at -35°C during transportation to the laboratory.

References

[1] Datasheet of Picarro G4301 GasScouter, available at https://www.picarro.com/support/library/documents/gasscoutertm_g4301_analyzer_datasheet (accessed 1 Nov 2023).

[2] McAuliffe, C. "Gas chromatographic determination of solutes by multiple phase equilibrium." *Chem. Technol.* **1**, 46-51. (1971).

[3] Sawamoto, T., Kusa, K., Hu, R., & Hatano, R. Dissolved N2O, CH4, and CO2 in pipe drainage, seepage, and stream water in a livestock farm in Hokkaido, Japan, *Soil Sci. Plant Nutr.* **48:3**, 433-439. (2002).

[4] Konya, K., Iwahana, G., Sueyoshi, T., Morishita, T. and Abe T. Methane flux around the Gulkana Glacier terminus, Alaska summer 2019. *Polar Data Journal* **6**, 32-42. doi:10.20575/00000040 (2022).
